# Automatic Sensory Predictions: A Review of Predictive Mechanisms in the Brain and Their Link to Conscious Processing

**DOI:** 10.3389/fnhum.2021.702520

**Published:** 2021-08-18

**Authors:** Ruxandra I. Tivadar, Robert T. Knight, Athina Tzovara

**Affiliations:** ^1^Institute of Computer Science, University of Bern, Bern, Switzerland; ^2^Helen Wills Neuroscience Institute, University of California, Berkeley, Berkeley, CA, United States; ^3^Department of Psychology, University of California, Berkeley, Berkeley, CA, United States; ^4^Sleep-Wake Epilepsy Center | NeuroTec, Department of Neurology, Inselspital, Bern University Hospital, University of Bern, Bern, Switzerland

**Keywords:** prediction error, mismatch negativity, coma, sleep, anesthesia, P300

## Abstract

The human brain has the astonishing capacity of integrating streams of sensory information from the environment and forming predictions about future events in an automatic way. Despite being initially developed for visual processing, the bulk of predictive coding research has subsequently focused on auditory processing, with the famous mismatch negativity signal as possibly the most studied signature of a surprise or prediction error (PE) signal. Auditory PEs are present during various consciousness states. Intriguingly, their presence and characteristics have been linked with residual levels of consciousness and return of awareness. In this review we first give an overview of the neural substrates of predictive processes in the auditory modality and their relation to consciousness. Then, we focus on different states of consciousness - wakefulness, sleep, anesthesia, coma, meditation, and hypnosis - and on what mysteries predictive processing has been able to disclose about brain functioning in such states. We review studies investigating how the neural signatures of auditory predictions are modulated by states of reduced or lacking consciousness. As a future outlook, we propose the combination of electrophysiological and computational techniques that will allow investigation of which facets of sensory predictive processes are maintained when consciousness fades away.

## Introduction

Learning information from our environment and forming predictions about future events is a key skill for survival. Stimuli from the world around us contain repetitive rules and patterns, as for example music, or speech. Being able to form predictions about future events facilitates perception and increases chances of survival, as a deviation from an expected pattern can signal danger.

**FIGURE 1:**
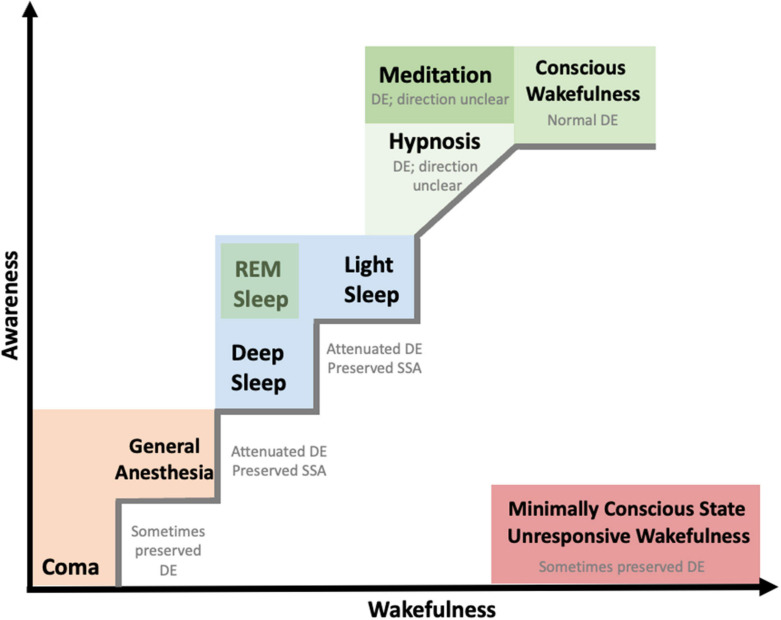
Manifestation of auditory predictive processes in different consciousness states. States of consciousness are placed according to the observed level of wakefulness and awareness, adapted from Laureys, 2005. The colors represent similarities or overarching groupings of states of consciousness. While there is a continuum from coma to conscious wakefulness, minimally conscious state and unresponsive wakefulness syndrome do not lie on the same continuum; neither do REM sleep, meditation, and hypnosis. The positioning of different consciousness states is following previous studies (e.g., Laureys, 2005) when possible and is an approximate estimate for consciousness states that were not originally included in previous studies (e.g., hypnosis, meditation). *DE: deviance effects, grouping together mainly effects observed at scalp EEG level. For more fine-grained information for each consciousness state we refer readers to Tables 1–4.

The human brain has the astonishing capacity to formulate predictions about future events, relying on internal models that generate automatic predictions (generative models) about the most plausible states of the environment given prior information. Neural predictions are generated not only in the case of active perception (SanMiguel et al., 2013), but also when conscious access to the environment is diminished, such as in sleep, anesthesia, or coma (Figure 1). The study of predictive processes has pervaded neuroscientific publications in the last three decades and painted a new view of the brain as a predictive organ (Dayan et al., 1995; Friston, 2005). Prediction provides explanation of phenomena at both the macro- and the micro-scales of brain functioning, including psychology (perception, cognition) and electrophysiology (neuronal processes). The study of predictive processes, which was first hinted to in the later 1800s (Lotze, 1852; Von Helmholtz, 1867) has been concretely formalized by statistics, information theory and machine learning. This review will focus on how sensory predictions have been used to probe different states of consciousness, and on what unknowns they have revealed about brain dynamics and functioning in these different states. By summarizing research done in both humans and animals, we examine the different components of the predictive network, and how these are modulated by conscious perception.

## Introduction to Auditory Predictive Mechanisms

### Paradigms for Studying Sensory Predictions and Consciousness

The most common sensory modality for studying predictive processes in the absence of consciousness is the auditory modality. Auditory stimulation is relatively straightforward to achieve, and it can reach the brain even in the absence of attention, or under conditions where eyes are closed, such as for example during sleep. The most famous paradigm for studying sensory predictions is the oddball paradigm (Näätänen and Alho, 1995; Garrido et al., 2009b), where a stream of identical repeated sounds (standards) is broken by an oddball, or a different sound (deviant), that is occasionally presented. In this paradigm, a regularity is built by repeating stimuli or sequences of stimuli. Prediction errors (PE) are signaled when deviations from the established regularity occur (Mumford, 1992; Rao and Ballard, 1999; Friston, 2005), by comparing neural responses to predicted (standard) versus observed (deviant) stimuli. Other experimental paradigms consist of the roving paradigm, where the oddball sound is followed by a stream of identical sounds, which at some point become standards, and are then followed by another stream of oddball sounds that turn into standards, with this pattern repeating (Garrido et al., 2009b); and the local-global paradigm (Bekinschtein et al., 2009), which is used to study local and global deviance effects. In the local-global paradigm, two forms of regularities are created – a local and a global one. These two types of regularities are built over single sounds (local), or groups of sounds (global). For local deviance, a standard sound is repeated a few times, followed by a deviant sound (e.g., aaaaB). This is similar to the deviance effect in a standard oddball paradigm. The global deviance effect is built by repeating this classic oddball structure, and then breaking this sequence by replacing the deviant in the third repetition with a standard (aaaaB aaaaB aaaaa).

### Mechanisms Underlying Auditory Predictions

#### Stimulus Specific Adaptation and Deviance Detection

To formulate a prediction, first a regularity needs to be established, often through repetition. Repeating a given stimulus, for example a sound, results in a reduced response at the neural level, commonly referred to as stimulus specific adaptation (SSA) in animal research (Carbajal and Malmierca, 2018; Sikkens et al., 2019), and repetition suppression (RS) in human research (Rangarajan et al., 2020). SSA quantifies the change in the firing rate of a neuron when a certain tone is frequently or infrequently presented (Ulanovsky et al., 2004; Khouri and Nelken, 2015). The SSA was first recorded in the cortex of anesthetized cats (Condon and Weinberger, 1991), where small but precise reductions in the responses to standard, tones were demonstrated, appearing minutes after the first presentation of the standard, and lasting for an hour or more. Neurons along the auditory pathway and in frontal and subcortical areas (see section “Cortical and Subcortical Generators”) show progressively reduced responses to repetitive stimuli, possibly as a result of short-term plasticity (Carbajal and Malmierca, 2018). Interestingly, neurons along the auditory processing pathway can express SSA, which in mice include parts of the inferior colliculus (IC), the dorsal and medial divisions of the medial geniculate body (MGB) and parts of the auditory cortex (Carbajal and Malmierca, 2018). This pathway is thought to carry predictions and prediction error signals (Carbajal and Malmierca, 2018).

A second crucial component of formulating a prediction is being able to detect violations from an established regularity. A deviant event may result in an increased neuronal response compared to the response to regular events, a phenomenon referred to as Deviance Detection (DD; Sikkens et al., 2019). For DD to occur, the increased neural response to deviant stimuli needs to be stronger than the neural response to standard stimuli, over and above SSA. DD is considered a correlate of error signaling (Sikkens et al., 2019). Although SSA occurs at early latencies, generally within the first 80 ms after stimulus onset, DD occurs at later latencies, around 120–240 ms post-stimulus onset (Sikkens et al., 2019). Macroscopically, these two processes of SSA and DD are thought to be contributors to a human EEG signature of regularity detection, the MMN (Sikkens et al., 2019).

#### Mismatch Negativity

The Mismatch Negativity (MMN) signal was first discovered in the late 1970’s (Näätänen et al., 1978). The MMN manifests as a negative component of a *difference wave* peaking at about 100–250 milliseconds (ms) post-deviance onset, obtained by subtracting responses to standard stimuli from responses to deviant stimuli (Näätänen, 2003; Garrido et al., 2009b). MMN was originally thought to be elicited based on a previously created sensory memory trace (Näätänen, 2003), thus offering an observation window into the central auditory system and its functioning (Näätänen and Escera, 2000). This is known as the “trace-mismatch” explanation of MMN (Winkler, 2007), where MMN is seen as a signal of mismatch or surprise between a *retrospective* memory trace and the current input. Another interpretation of MMN is found in the adaptation hypothesis (May et al., 1999; Jääskeläinen et al., 2004). According to this hypothesis, cells tuned to standard sounds adapt, while cells tuned to more infrequent deviant sounds do not adapt and thus elicit higher responses (May et al., 1999). More recently, the MMN has been examined under the lens of predictive coding, which suggests that the MMN is a neural signature of a sensory prediction error signal, and that it represents an ‘error’ response that is elicited by deviant sounds (Garrido et al., 2009b). This view is supported by computational modeling studies, which have linked trial by trial changes in the MMN signal with the adjustment of an internal probabilistic model of the environment (Lieder et al., 2013). Under predictive processing, MMN is a signal of mismatch between sensory input and, contrary to the “trace-mismatch” hypothesis, a *prospective* and not retrospective sensory stimulus.

Interestingly, the MMN is described as a pre-attentive, automatic response, which can be elicited despite variations in states of wakefulness (Sculthorpe et al., 2009), such as during sleep or anesthesia, coma, or states of altered awareness, including hypnosis and meditation (Cahn and Polich, 2009; Chennu and Bekinschtein, 2012; Morlet and Fischer, 2014; Jamieson, 2016). In addition to extensive research in humans, MMN responses have also been recorded in cats (Csépe et al., 1987; Pincze et al., 2001), monkeys (Javitt et al., 1992, 1994), rabbits (Ruusuvirta et al., 1995, 1996a, b), guinea pigs (Kraus et al., 1994), and rats (Shiramatsu et al., 2013; Harms et al., 2014), via epidural EEG electrodes or cortical surface microelectrode arrays. Results are comparable, but not completely identical. For example, MMN responses in cats appear with shorter latencies due to the smaller size of cat cortex (Pincze et al., 2001). In summary, the MMN is an event-related potential (ERP) component that represents a scalp EEG signature of predictive processing, and is observed across species and states of consciousness.

#### P300

The P300 component is a positive deflection in the human ERP, with a peak latency at about 300 ms post-stimulus onset in response to a novel or task-relevant stimulus (Sutton et al., 1965). The P300 is usually elicited in an oddball paradigm when behavioral responses to deviants are demanded – thus, as a response to a target deviant stimulus (Picton, 1992). It has been proposed that the P300 reflects contextual updating (Donchin, 1981; Donchin and Coles, 1988) driven by attentional processes (Polich, 2007), namely updating of a stimulus or of task-related (working) memory and expectancies (Verleger, 1988). The P300 has two main subcomponents, the P3a and P3b, which have different topographies and functional implications. While the P3a is fronto-centrally distributed and appears as a response to novel and distracting stimuli, the P3b is maximal over parietal recording sites in response to conscious detection of target and novel stimuli (Squires et al., 1975; Polich, 2007).

### Neural Circuits Underlying Auditory Predictions

Predictive neural traces manifest in multiple stages of sensory processing. The most prevalent view is that higher level regions in a processing hierarchy generate and propagate sensory predictions to lower level regions, which compare these predictions to the actual sensory input (Rao and Ballard, 1999; Friston, 2005). Predictions flow ‘down’ the processing stream from higher level areas to lower level areas, while the opposite is true for error signaling, meaning that errors are signaled ‘upward’ by lower level areas detecting a mismatch with the current prediction (Rao and Ballard, 1999; Bastos et al., 2012). Importantly, the signaling of predictions and errors is posited to underlie multiple stages of information processing, so that sensory processing would, at each processing level, have to resolve the correspondence between predictions and sensory input (Friston, 2005; Summerfield and Egner, 2009). For this reason, some argue that predictive coding theories go beyond the standard bottom-up and top-down dichotomy (Rauss and Pourtois, 2013), as higher levels do not only modulate activity at lower levels of processing, but have the chance to initiate such activity (Mumford, 1992), in addition to lower level stages of the hierarchy being able to generate predictions for higher-level error signals (Kok and de Lange, 2015). There are multiple models of predictive processing (e.g., Spratling, 2008a, b; Bastos et al., 2012), which deviate from standard models with regards to where the error units are situated (i.e., in middle and not superficial cortical layers), and how predictions flow (i.e., not only ‘downward’ through the processing stream, but also ‘upward’). Nevertheless, most models posit that error and predictive units have different laminar profiles (see Heilbron and Chait, 2018 for a detailed review).

#### Cortical and Subcortical Generators of Sensory Predictions

Sensory predictions are supported by distributed circuits in the brain, including sensory and prefrontal, but also subcortical regions, which may compute different variables related to predictions (Johnson et al., 2020). Predictive mechanisms are not only inherent properties of microcircuits in the brain, but also find expression through cortical connectivity (Johnson et al., 2020). Connected regions in the cortical hierarchy interact recurrently in a joint effort to find the world model that best explains the sensory inputs in the prediction units, and thereby reduce the activity of these units (Kok and de Lange, 2015).

In the auditory modality, magnetoencephalography (MEG) studies first showed that the MMN is generated in the auditory cortex (Hari et al., 1984). Later, using functional Magnetic Resonance Imaging (fMRI) and EEG, it was discovered that frontal regions are also involved in MMN generation (Alho, 1995; Opitz et al., 2002). Specifically, Opitz et al. (2002) used fMRI and EEG to study the temporal and frontal generators of the MMN and showed that responses to deviant stimuli of medium and large, but not small amplitude are found in the superior temporal gyrus (STG) bilaterally, and in the inferior frontal gyrus (IFG). Since then, these areas were often studied using EEG and fMRI combined with dynamic causal modeling (Garrido et al., 2007, 2008, 2009a; Boly, 2011; Chennu et al., 2016), and were also confirmed by multiunit activity (MUA) recordings (Nieto-Diego and Malmierca, 2016) and local field potential (LFP) measurements of SSA in rats (Imada et al., 2013). The neural correlates of the P300 component have been localized to multiple brain regions. The generators of the P3a include frontal cortical generators, the cingulate cortex, the supramarginal gyrus, and the hippocampus, while the generators of the scalp P3b include mainly temporo-parietal and frontal regions (Fonken et al., 2019).

Intracranial EEG (iEEG) recordings in humans have further advanced our understanding of the neural underpinnings of the predictive circuit (Johnson et al., 2020), by confirming the involvement of temporal and frontal regions in responding to deviant events (e.g., Rosburg et al., 2005). Additionally, Durschmid and colleagues showed that the temporal cortex detects deviations at the level of single stimuli, while prefrontal regions are sensitive to whether these deviations were predictable (Dürschmid et al., 2016), as well as to the likelihood of a deviant sound to occur (Dürschmid et al., 2019). Intracranial recordings have also implicated the posterior cingulate and parietal lobe (Halgren et al., 1995; Clarke et al., 1999), limbic structures such as the hippocampus, the amygdala (Halgren et al., 1980), and basal ganglia and thalamic circuits such as the caudate nucleus (Kropotov et al., 2000) and nucleus accumbens (Zaehle et al., 2013; Dürschmid et al., 2016) in supporting the auditory predictive network.

In addition, Cacciaglia et al. (2015) used event-related fMRI during an oddball task and found evidence of involvement of human inferior colliculus (IC) and MGB of the thalamus (Cacciaglia et al., 2015), confirming previous similar results found using SSA in animals for the occurrence of infrequent speech-like stimuli (Kraus et al., 1994), as well as for sounds with different binaural phases (King et al., 1995). fMRI studies further involved the amygdala (Kropotov et al., 2000; Czisch et al., 2009; Blackford et al., 2010) and hippocampal (Blackford et al., 2010) structures in deviance detection. Subsequent single unit recordings, and fMRI implicated the IC (Pérez-González et al., 2005; Malmierca et al., 2009; Patel et al., 2012; Gao et al., 2014) and the MGB (Anderson et al., 2009; Antunes et al., 2010; Richardson et al., 2013) in SSA (see also, Duque et al., 2015 for an extensive review on subcortical structures implicated in SSA generation).

In summary, sensory predictions rely on a distributed network of brain regions, expressed in low-level sensory processing areas, cortico-thalamic circuits involving subcortical thalamic and basal ganglia structures together with the amygdala and hippocampus, as well as higher-level parietal and frontal areas. This complex distributed network involved in sensory processing and PE generation works in concert to allow learning of sensory regularities and the formation of predictions.

### Attention

The role of attention in MMN generation was initially investigated in auditory tasks, where the ear to be attended was manipulated (Näätänen et al., 1993; Trejo et al., 1995; Alain and David, 1997). The debate was initiated when Näätänen proposed that the MMN was unaffected by manipulations of attention (see Näätänen, 1990 for a review). This view was challenged by research showing attentional effects on MMN (Woldorff and Hillyard, 1991; Szymanski et al., 1999; Auksztulewicz and Friston, 2015). There is now a plethora of studies showing that attention enhances the amplitude of MMN (Woldorff and Hillyard, 1991; Alain and David, 1997; Szymanski et al., 1999; Chennu et al., 2013; Auksztulewicz and Friston, 2015) and P300 (Chennu et al., 2013) responses. Electrophysiologically, manipulations of attention have been shown to predominantly affect the detection of oddball stimuli in prefrontal, but not temporal, regions, and to increase effects of oddball detection (Kam et al., 2020).

Later views suggested that the MMN response can be considered as a two-step process, composed of both standard formation and deviance detection (Sussman, 2007). The standard formation phase consists of auditory processes such as scene analysis and is susceptible to attentional effects. In contrast, the deviance detection phase, which depends on the standard formation phase, is independent of attentional manipulations. From a computational perspective, attention is thought to increase the precision of PEs, leading to more reliable estimates and a more accurate update of an environmental model (Auksztulewicz and Friston, 2015).

Although attention is not the focus of the present review, it can be argued that inattentive states represent states where sensory signals and predictions are elicited in an automatic way, as in unconscious states. We therefore mentioned these key findings in the field to emphasize that the brain not only produces predictions about the features of a signal (i.e., intensity, frequency, etc.), but also about the signal’s reliability or precision (i.e., how predictable is the signal). When signal reliability is low, such as in inattentive conditions, deviations are down-weighted; when it is high, deviations are amplified and prioritized for further processing (Heilbron and Chait, 2018). In this view, predictive processes and attentive processes are distinct, independent processes which can interact. The role of predictive mechanisms is making inferences about what causes the sensory input and how precise this input is, whereas attention optimizes the precision of this input and regulates the gain of feedforward PEs (Schröger et al., 2015).

## Sensory Predictions in Reduced Consciousness States

Automatic sensory predictions manifest during wakefulness, but also when conscious access to the environment is lost, as will be subsequently reviewed. The interest for studying how neural responses are elicited during various awareness states first appeared when it was discovered that the MMN was evoked in the absence of attention (Näätänen, 1990), albeit with a much lower amplitude. MMN responses were even observed when subjects were engaged in other tasks, such as reading a book (Näätänen et al., 1993). Early studies recording MMN responses in animals anesthetized with barbiturates also confirmed MMN responses (Csépe et al., 1987; Javitt et al., 1992; Kraus et al., 1994). MMN responses were also observed during sleep in humans (Nielsen-Bohlman et al., 1991) and animals (Csépe et al., 1987). These studies indicated a great potential for studying auditory predictions in the absence of conscious access to the environment. Therefore, the value of the MMN response as a diagnostic tool for patients with disorders of consciousness (Chennu and Bekinschtein, 2012), or with psychiatric disorders (e.g., depersonalisation and derealisation) became evident (Lew et al., 2003; Kotchoubey et al., 2005; Wijnen et al., 2007).

Understanding the neural underpinnings that are associated with the emergence of conscious experience is of one of the main unresolved questions in neuroscience, with a first major challenge consisting in the clarification of the experimental definition of the term consciousness (Dehaene and Changeux, 2011). This is a fundamental challenge, due to the implications it brings for patients in coma, anesthesia, and those suffering from disorders of consciousness. Here, we adopt a widely used, non-exhaustive, functional definition of consciousness, which assesses conscious states by their expressed level of consciousness (wakefulness) on the one hand, and content of consciousness (awareness) on the other hand (Laureys, 2005; and Figure 1). This clinical definition of consciousness is also used to diagnose disorders of consciousness (see Giacino et al., 2014 for a review), characterized by a disrupted relationship between awareness and wakefulness (Gosseries et al., 2011), where observations of spontaneous and stimulus-evoked behaviors are used. Predictive processing was recently characterized as a “neural motif,” which is present in many computations in the brain (Aitchison and Lengyel, 2017), but how does it relate to our conscious wakefulness and awareness? In fact, auditory predictive coding is commonly used to assess residual brain functions in patients with disorders of consciousness, often through scalp EEG components that are considered as neural signatures of predictive processing (Chennu and Bekinschtein, 2012; Gosseries et al., 2014a).

In the next sections we will provide an overview of findings from the last 30 years studying the extent to which the neural markers of predictive processes are altered by reduced or absent consciousness. We will present findings from studies in sleep, anesthesia, disorders of consciousness, or altered states of consciousness, in humans and animals. In particular, we will focus on different neural signatures of auditory predictive processes, such as MMN and P3, or SSA, and we will review how these are modulated by the absence or reduction of consciousness. When possible, we will elaborate on neural mechanisms and circuits of auditory predictions, for example, in the case of studies using techniques with a high spatial resolution (e.g., iEEG or source localization techniques). In other cases, we will discuss findings based on neural markers of predictive processing at a more macroscopic level such as scalp EEG components and their possible clinical applications.

## Sleep

Sleep represents a naturally occurring and rapidly reversible state of reduced consciousness (Campbell and Colrain, 2002). Sleep electrophysiology is altered with respect to wakefulness (Destexhe et al., 2007; Cox et al., 2014), but is well-characterized and relatively uniform across individuals (Steriade, 2006). In terms of the physiology of sleep, we distinguish paradoxical sleep or rapid eye-movement sleep (REM), and non-REM (NREM) sleep, which is further divided into three stages. NREM1 is the sleep onset period, NREM2 is light sleep, and NREM3 sleep is slow-wave-sleep. Different sleep stages have been associated with reduced consciousness or arousal (Goupil and Bekinschtein, 2012; Lendner et al., 2020).

### Research in Humans

Several studies have investigated the neural correlates of MMN during sleep (Camman et al., 1987; Csépe et al., 1987; Näätänen and Lyytinen, 1994; Sallinen et al., 1994; Winter et al., 1995; Loewy et al., 1996; Atienza et al., 1997; Sallinen and Lyytinen, 1997; to name a few). After the wave of research in the 90’s, which employed standard intensity or duration oddball paradigms, the consensus was that MMN and P300 components appeared in REM sleep, but not in NREM2 (see e.g., Winter et al., 1995; Loewy et al., 1996, 2000; Cote, 2002; Colrain and Campbell, 2007; Sculthorpe et al., 2009). The main evoked potentials were K-complexes and late potentials that were functionally different from the classic deviance response (Wesensten and Badia, 1988; Nielsen-Bohlman et al., 1991; Van Sweden et al., 1994; Nordby et al., 1996). Nevertheless, some studies still indicated differential processing of auditory information even during deeper sleep stages (Nielsen-Bohlman et al., 1991; Winter et al., 1995). Laboratories therefore modified their paradigms in order to have more sensitive tests, and presented either rapidly succeeding stimuli (every 150 ms) (Sabri et al., 2003), or used “hyper-salient” stimuli (Chennu and Bekinschtein, 2012) – i.e., very rare, very deviant stimuli, as used for example by Loewy and colleagues, with low probability and 1000 Hz difference between the standard and the deviant stimuli. In some of these studies, MMN responses were elicited during NREM1 and NREM2 (Sabri et al., 2003; Sabri and Campbell, 2005), whereas in others they were only evoked during REM sleep (Loewy et al., 1996; see Table 1A for a summary).

**TABLE 1:** Studies in sleep.

(A) Humans			

Study	Paradigm	Phase	Deviance effects
Wesensten and Badia, 1988	Pitch oddball	REM	Yes
		NREM2	Yes
Nielsen-Bohlman et al., 1991	Pitch oddball	NREM2	Yes
Van Sweden et al., 1994	Pitch oddball	REM	Yes
		NREM	Yes
Winter et al., 1995	Pitch oddball	NREM	Yes
Nordby et al., 1996	Pitch oddball	REM	No
		NREM	No
Loewy et al., 1996	Pitch oddball	REM	Yes
		NREM 2	No
		NREM 3	No
Loewy et al., 2000	Intensity oddball	REM	No
		NREM 2	No
Sabri and Campbell, 2002	Pitch oddball	NREM 3	Yes
Sabri et al., 2003	Pitch oddball	NREM 2	Yes
		NREM 1	Yes
Sabri and Campbell, 2005	Pitch oddball	REM	Yes
		NREM 1	Yes
		NREM 3	Yes
Sculthorpe et al., 2009	Repetition oddball	REM	Yes
		NREM	No
Strauss et al., 2015	Local-global	REM	Only local
		NREM 1	Only local
		NREM 2	Only local

(B) Animals				

Study	Paradigm	Phase	Deviance effects	Species
Csépe et al., 1987	Pitch oddball	NREM	Yes	Cats
Nir et al., 2015	Pitch oddball	REM	SSA	Rats
		NREM	SSA	

*NREM, non-rapid eye movement sleep; REM, rapid eye movement; SSA, stimulus-specific adaptation.

A more recent study employed MEG and EEG recordings during sleep and used a local-global paradigm (Strauss et al., 2015). Results showed a disrupted global response in NREM2 sleep, associated with an absence of the P300 response together with a simultaneous absence of behavioral responses, despite retained local mismatch responses across all sleep stages (Strauss et al., 2015). Moreover, authors used an additional manipulation where expectation was induced by alternating different sounds (aBaBa and aBaBB sequences), instead of repeating the same stimulus (aaaaa). In this case, the differential response that was observed between predicted and unpredicted sequences during wakefulness vanished during NREM2 sleep. This was interpreted as evidence that predictive processing during sleep could be explained with an adaptation framework (through repetition of the same stimuli) and not by using prediction error (through repetitions of different stimuli) mechanisms.

Even when MMN responses are present during sleep, their characteristics (i.e., amplitude or latency) are typically attenuated with respect to awake conditions (Atienza et al., 2001). It is, however, unclear whether predictive processes during sleep are altered because the underlying predictive computations are fundamentally different compared to wakefulness, or because the sleep electrophysiology is modified (Sabri and Campbell, 2002). Apart from detecting deviant events, there is an ongoing debate whether new information can be learned during sleep, and if so, under which conditions (Andrillon et al., 2017). A large body of literature reports no evidence for learning new rules in deep NREM sleep, but more recent findings show that semantic associations can be learned if these are presented during peaks (i.e., “up” states) of slow-wave activity (Züst et al., 2019), which are characterized by similar conditions of cortical excitability as wakefulness (Destexhe et al., 2007; Cox et al., 2014). Moreover, other studies have shown that sleep facilitates encoding of previously learned generative models, improving sensory predictions (Lutz et al., 2018).

### Research in Animals

Animal sleep research has investigated evoked responses in sensory systems (Hennevin et al., 2007). From a physiological viewpoint, two states of sleep are classically categorized in animals, paradoxical or REM sleep, and NREM sleep. Physiological studies in sleep further demonstrate preserved auditory processing (Edeline et al., 2000; Issa and Wang, 2008), with reported decreases in the amount and quality of information reaching the higher-level cortices (for an extensive review, see Coenen and Drinkenburg, 2002; see also Murata and Kameda, 1963). This reduction in information transmission is thought to be due to thalamic gating (McCormick and Bal, 1994), with sensory input to the cortex partially blocked at the thalamus (Hall and Borbely, 1970; Edeline et al., 2000). Pre-thalamic processing is thought to be *mostly* maintained (Steriade, 1991). Nevertheless, relevant stimuli can have some form of impact on the functional state of the sleeping animal, suggesting that the sleeping brain is still able to judge the meaningfulness of stimuli (Nielsen-Bohlman et al., 1991). Sophisticated paradigms suggest that simple forms of learning are also still possible, as for example in extinction (where a pre-conditioned association between two stimuli is erased) and pre-exposure (when animals are exposed to the to-be-conditioned stimulus before actual conditioning) experiments; and there is evidence that new associations can be formed with lower quality than the ones formed during waking (Coenen and Drinkenburg, 2002).

**FIGURE 2:**
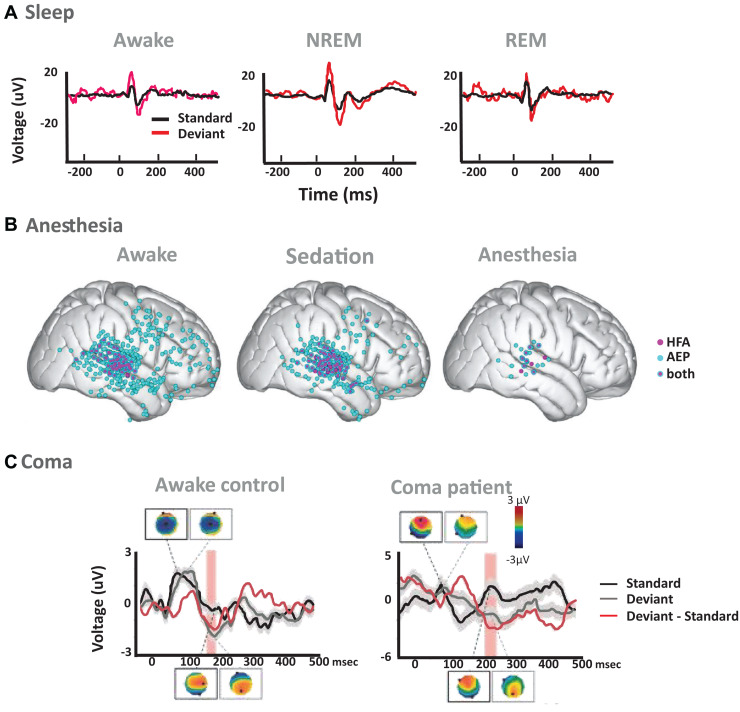
Examples of auditory predictive processes across states of reduced consciousness. **(A)** Auditory averaged ERP responses to standard (black) and deviant (red) tones during normal wakefulness (left), NREM sleep (middle) and REM sleep (right) from EEG recordings in rats. Figure adapted from Nir et al. (2015). EEG recordings showed weaker responses following standard compared to deviant tones in wakefulness, NREM and REM sleep, an effect that was additionally quantified by the authors as SSA in single unit activity of the primary auditory cortex (Nir et al., 2015). **(B)** Local Deviance effects in electrocorticography (ECoG) recordings of patients with epilepsy (Figure adapted from Nourski et al., 2018). Pink dots show electrodes with significant differences between responses to standard and deviant sounds in high frequency activity (HFA; 70–150 Hz); blue dots show electrodes with differences in evoked potentials (AEP); and pink and blue dots show electrodes with significant AEP and HFA effects. Local deviance was defined as significant increases in response to the deviant versus standard stimuli along a 0–800 ms post 5th stimulus window: (aaaaB – aaaaa) or (BBBBa – BBBBB). Stimuli were vowels /α/ and /i/, extracted from a female voice uttering the words h/α/d and h/i/d. Significant electrodes are shown for the awake state (left), for sedation (middle) and for the anesthesia state (right). **(C)** Auditory evoked potentials (AEP) and scalp topographies for an exemplar awake control and a coma patient, measured over frontal electrodes to standard (black) and duration deviant (gray) sounds, as well as the difference of the two responses (red; Figure adapted from Tzovara et al., 2013). The awake control shows a typical N100 response to auditory stimuli, manifesting as a central negativity in the AEP topography, and an MMN response starting around 170 ms post-stimuli onset. The exemplar patient shows differential responses between standard and deviant sounds at later latencies, after 220 ms post-stimuli onset. Red rectangles signify periods of significant difference in response to standard vs. deviant sounds.

An early study in cats reported that the MMN can be elicited during all sleep stages (Csépe et al., 1987). Auditory evoked potentials were elicited by standard and deviant tones of different probabilities during wakefulness and sleep. A large MMN response was elicited by deviant tones, with MMN amplitude inversely proportional to deviants’ probability. MMN during slow-wave sleep exhibited longer latency and was only evoked by deviant tones at the lowest probabilities. Another more recent study in rats used an oddball paradigm and found comparable SSA responses across REM, NREM and wake cycles in the core auditory region, defined by the authors as the core auditory fields receiving input from the ventral division of the medial geniculate nucleus of the thalamus (Nir et al., 2015; Figure 2B; see also Table 1B, for a summary). This suggests that evoked activity in low-level sensory cortices during sleep is driven by external stimuli with little modulation by the vigilance state, and that the disconnection of cortical processing during sleep may occur at a later stage, thus corroborating the physiological findings described above.

### Conclusion

In conclusion, the majority of sleep studies suggest that auditory predictive processing may be retained during sleep, in particular within core auditory areas (Nir et al., 2015). There is consensus that scalp EEG components related to predictive processes can manifest during REM sleep, with similar characteristics as during wakefulness. For NREM, the question of whether auditory predictions can occur remains actively debated. One key factor that will need to be taken into account in the design of new experiments and during data analysis is the complex and dynamic brain physiology of sleep.

Different sleep stages are characterized by multiple local disruptions (Drummond et al., 2004; Magnin et al., 2010), leading to qualitatively different epochs with differences in sensory processing (Hennevin et al., 2007). Additionally, different stages of sleep are not homogeneous, as they are characterized by tonic and phasic fluctuations of arousal, of the background EEG activity, and of neuromodulator release (Hobson et al., 2000). As a result, cortico-thalamic long-range connectivity is affected, while some basic cortico-cortical connectivity might be preserved, as for example in the default mode network (Koike et al., 2011).

These fluctuations in sleep physiology might explain the attenuated MMN responses measured during sleep, and might mirror the decreasing thalamic activity, by indicating an impaired bottom-up component of MMN elicitation (Atienza et al., 2002). The impaired top-down component might stem from prefrontal lobe deactivation during sleep (Atienza et al., 2002). The cortico-thalamic network during REM sleep seems to be characterized by general activations in thalamic and posterior areas including temporo-occipital cortices (Maquet et al., 1996; Braun et al., 1997; Maquet, 2000; Portas et al., 2000), while frontal area activity is reduced (Maquet, 2000; Portas et al., 2000). All these areas are deactivated during NREM sleep (Maquet, 2000). Alternatively, connectivity at a later stage of information processing has also been reported during sleep (Massimini et al., 2005; Horovitz et al., 2009; Tagliazucchi et al., 2013), with preserved activation of primary sensory cortices in both animals (Peña et al., 1999; Edeline et al., 2001; Issa and Wang, 2008) and humans (Portas et al., 2000; Czisch et al., 2002; Dang-Vu et al., 2011).

Future research investigating predictive processing in sleep is crucial, given the sparseness of the current literature. Auditory paradigms are particularly important for assessing brain processing during sleep, as well as associations between sleep disorders and generalized reduced cognitive performance (Pilcher and Huffcutt, 1996; Banks and Dinges, 2007), or impaired auditory processing (Raz et al., 2001; Key et al., 2009; Bortoletto et al., 2011; Liberalesso et al., 2012; Leite et al., 2017).

## Anesthesia

Phenomenologically and behaviorally, anesthetic states can be described as a continuum ranging between mild sedation, “a pharmacologically induced, reversible state, characterized by dose-related impairment of cognitive functions, including attention and memory, but during which consciousness and awareness are maintained” (Stamatakis et al., 2010), to complete anesthesia, “a drug-induced loss of consciousness during which patients are not rousable, even by painful stimulation” (Anesthesiologists Task Force on Intraoperative Awareness, 2006).

Anesthetics have complex effects on neural activity, such as alterations in the activity of wide-spread cortico-thalamic networks (Rudolph and Antkowiak, 2004; Scheinin et al., 2021), and disruptions of cortico-thalamic connectivity (Guldenmund et al., 2017). Interestingly, general anesthesia and NREM sleep share similarities, such as slow oscillatory activity, a disruption of cortico-cortical connections (Massimini et al., 2005; Pal et al., 2016), and changes in non-oscillatory neural dynamics (Lendner et al., 2020). During anesthesia and NREM, thalamocortical hyperpolarized neurons are alternating between active and silent periods. By contrast, during wakefulness and REM sleep, the thalamocortical system is depolarized with awake-like low-voltage activity, and with tonic firing in neurons (Steriade et al., 2001). At high doses, general anesthesia during surgery can approximate brain stem death, where patients are unconscious, have inhibited brain stem reflexes, do not respond to nociceptive stimuli, and require cardiorespiratory and thermoregulatory support (Brown et al., 2010). These levels of anesthesia can be accompanied by isoelectric (i.e., almost a flat line) EEG activity (Brown et al., 2010).

In terms of cerebral metabolism, most anesthetics result in a general reduction in cortical brain activity, with certain regions, including cortical association areas, the thalamus, and the midbrain showing a higher decrease in cerebral metabolism (Heinke and Koelsch, 2005). In human studies, anesthesia is typically induced using either propofol (Plourde and Picton, 1991; Reinsel et al., 1995; Koelsch et al., 2006) or opioids (Plourde and Boylan, 1991). Propofol is an agonist at the GABA receptor and exerts a hypnotic and sedative effect through this mechanism (Rudolph and Antkowiak, 2004). Light propofol anesthesia, as administered in surgery, causes stage 2 sleep-like brain electrophysiological activity, with sleep and sleep-like spindles appearing during deep propofol anesthesia (Stamatakis et al., 2010; see Purdon et al., 2015, for a review). Opioids such as fentanyl are mostly used in cardiovascular surgery due to limited fluctuations in cardiovascular dynamics (Saidman et al., 1984). The EEG trace during opioid anesthesia is characterized by high amplitude slow delta waves (Wauquier et al., 1984). Opioids provide anesthesia, analgesia and unconsciousness after premedication with other anesthetic agents such as benzodiazepines (Sebel et al., 1981).

### Research in Humans

Early human anesthesia studies did not compute the MMN response, but rather examined the P300 response, due to its suspected association with conscious awareness (Plourde and Boylan, 1991; Plourde and Picton, 1991; Reinsel et al., 1995). These studies report a decrease in amplitude of the P300 response with progressive sedation and abolishment when unconsciousness is reached (Plourde and Boylan, 1991; Plourde and Picton, 1991; Sneyd et al., 1994; Reinsel et al., 1995), accompanied by absent behavioral responses to deviant stimuli (Plourde and Picton, 1991).

Later studies carried out in the 2000’s (Simpson et al., 2002; Yppärilä et al., 2002; Heinke et al., 2004; Koelsch et al., 2006) started to measure MMN responses alongside the P300 responses. These studies reported a dose-dependent incremental breakdown of MMN and P300 (Yppärilä et al., 2002; Heinke et al., 2004; Koelsch et al., 2006). As patients transition from wakefulness to anesthesia, AEPs tend to decrease in amplitude: Simpson et al. (2002) reported that N100 (thought to reflect the early processing of acoustic features of a stimulus; Näätänen and Picton, 1987) responses to auditory stimuli disappear when patients become unconscious, and MMN is no longer elicited right before consciousness is lost, at the point of highest propofol concentration at which patients are still responsive. Yppärilä et al. (2002) complemented these findings by showing that the amplitudes of AEPs including N100 and MMN gradually decrease, and latencies gradually increase as patients transition from light to deep sedation. Notably, a small subset of patients retains both MMN and P300 responses even in deep sedation (Yppärilä et al., 2002). Similar findings were reported by Heinke et al. (2004), who showed decreasing amplitudes and increasing latencies for MMN as propofol sedation progresses, and an abolishment of P300 responses in deeper sedation levels (Heinke et al., 2004).

Koelsch et al. (2006) measured MMN and P300 responses in healthy volunteers undergoing propofol sedation in a state of sedation shallower than surgical anesthesia, as participants were still arousable by loud and repeated utterances of their own name or by mild prodding. The authors noted reduced, but existent, MMN and P3a responses during propofol sedation, with a missing P3b response. With recovery from deep propofol sedation, MMN recovered quickly to wake levels, but not the P300 response. Lastly, Zhang et al. (2018) report that MMN is abolished during deep anesthesia. The authors used source localization techniques to investigate how the network underlying the MMN response during awake conditions is altered by anesthesia. Deviant stimuli during anesthesia induced less long-distance connections between frontal and temporal-parietal regions than in an awake state (Zhang et al., 2018).

More recent studies have employed the local-global paradigm (Shirazibeheshti et al., 2018; Witon et al., 2020) with high-density EEG or iEEG recordings (Nourski et al., 2018) to test this hypothesis directly. Specifically, Shirazibeheshti et al. (2018) measured high-density EEG during a local-global paradigm in wakefulness, propofol sedation, and recovery. During sedation, both local and global deviance responses were recorded, but their amplitude was reduced with respect to wakefulness. The authors observed an interaction between effects of local and global deviance, namely that effects of local deviance exacerbate effects of global deviance. Nevertheless, under anesthesia this interaction was reduced. The authors posited that the coincidence of local and global deviance had a facilitatory effect on global deviance responses, which was reduced when individuals were sedated. Witon et al. (2020) further examined the neural circuits of this effect and observed effects of sedation on local deviance responses during early (100–150 ms post-stimulus onset) and middle (250–350 ms) time periods, indicative of modulations of evoked power responses early in the processing pathway. The interaction between the local and global effects was significant in a late time window (400–600 ms). The authors found a locally mediated acceleration of global deviance responses (Witon et al., 2020) during sedation and recovery. The second important interaction – the local standard global deviant, representing the pure global deviance effect – was reduced in anesthesia compared to recovery. Here, deviance processing is thought to be instantiated by more higher-level than low-level predictions. Key findings during sedation included a reduction in amplitude of the responses, and a slowing of the responses to deviant stimuli, specifically in global deviance.

Nourski et al. (2018) examined the neural networks that are preserved for local and global deviance responses in iEEG recordings. High frequency activity responses, which correlate with local infragranular multi-unit activity and superficial dendritic potentials (Leszczynski et al., 2020), and intracranial auditory evoked potentials were recorded. Authors used vowels instead of pure tones in patients implanted in temporal and inferior frontal regions, as well as in the amygdala, under propofol sedation. This study reported retained local deviance effects under loss of consciousness in auditory regions, but not outside of these regions, indicating intact low-level sensory predictive processing independent of the state of consciousness (Figure 2). By contrast, local deviance responses in frontal regions began to reduce during initial sedation and vanished during anesthesia. Global deviance was completely abolished with anesthesia, and in some patients, it was abolished even during a sedated state in which they were still responsive (Nourski et al., 2018). The authors concluded that the presence of a global deviance effect is indicative of conscious processing, while its absence is not necessarily linked to loss of consciousness (see Table 2A for a summary).

**TABLE 2:** Studies in anesthesia.

(A) Humans					

Study	Paradigm	Agents	Anesthesia (A)/ sedation (S)	Surgical A/S	Deviance effects
Plourde and Picton, 1991	Pitch oddball	Thiopental, fentanyl, and isoflurane ± nitrous oxide	A	Yes	No
Plourde and Boylan, 1991	Pitch oddball	Sufentanil with lorazepam premedication	A	Yes	No
Reinsel et al., 1995	Pitch oddball	Propofol	S	No	Yes
Sneyd et al., 1994	Pitch oddball	Propofol	S	No	Yes
Simpson et al., 2002	Pitch oddball	Propofol	S - conscious	No	Yes
			S - unconscious		no
	Duration oddball		S - conscious		no
			S - unconscious		no
Yppärilä et al., 2002	Pitch oddball	Propofol	S	Yes	Yes
Heinke et al., 2004		Propofol	S - light	Yes	Yes
			S - deep		Yes
			S - unconscious		No
Koelsch et al., 2006	Pitch oddball	Propofol	S	No	Yes
Zhang et al., 2018	Pitch oddball	Propofol	S - deep	No	No
Nourski et al., 2018	Local-global	Propofol	A - conscious	Yes	Only local
			A - unconscious		Only local
Shirazibeheshti et al., 2018	Local-global	Propofol	S - unconscious	No	Local and global
Witon et al., 2020	Local-global	Propofol	S - moderate	No	Local and global

(B) Animals				

Study	Paradigm	Agents	Deviance effects	Species
Ruusuvirta et al., 1998	Pitch oddball	Urethane	Yes	Rats
Lazar and Metherate, 2003	Pitch-frequency oddball	Urethane-xylazine	SSA	Rats
Eriksson and Villa, 2005	Pitch oddball	Ketamine-xylazine	No	Rats
Astikainen et al., 2006	Pitch-intensity oddball	Urethane	Yes	Rats
Nakamura et al., 2011	Pitch-duration oddball	Fentanyl-medetomidine-isoflurane	Yes	Rats
Taaseh et al., 2011	Pitch oddball	Halothane	Yes	Rats
Xu et al., 2014	Aurality-specific noise	Sodium pentobarbital	SSA	Rats
Takahashi et al., 2015	Pitch oddball	Isoflurane	SSA	Rats
Ahnaou et al., 2017	Pitch oddball	Ketamine	Yes	Rats
Rui et al., 2018	Pitch oddball	Urethane	SSA	Rats
Ehrlichman et al., 2008	Pitch oddball	Ketamine	No	Mice
Chen et al., 2015	Pitch oddball	Isoflurane	SSA	Mice
Duque and Malmierca, 2015	Pitch oddball	Urethane ± acepromazine	SSA	Mice
Duque et al., 2018	Pitch oddball	Ketamine-xylazine	SSA	Mice
Lipponen et al., 2019	Duration oddball	Urethane	No	Mice
Uhrig et al., 2016	Local-global	Propofol	Only global, no local	Primates
		Ketamine	Only local, no global	
Kraus et al., 1994	Pitch oddball	Ketamine-xylazine	Yes	Guinea pigs
Christianson et al., 2014	Roving standard	Urethane-buprenorphine	SSA	Guinea pigs
Bäuerle et al., 2011	Roving standard	Ketamine-xylazine	SSA	Gerbils
Beckers and Gahr, 2012	Naturalistic oddball	Isoflurane	Yes	Songbirds

*SSA, stimulus-specific adaptation; Yes, effects other than SSA.

### Research in Animals

In animals, anesthesia is *mostly* induced using ketamine, urethane, or halothane (see Table 2B for summary). Anesthesia in general, whether with barbiturates or ketamine, seems to have more wide-spread effects in animals than in humans. Specifically, inhibition of auditory cortical units was reported 70 years ago (Thomas and Jenkner, 1952). Anesthetics are known to affect the entire central auditory pathway, from the dorsal cochlear nucleus (Young and Brownell, 1976) to core auditory regions (Gaese and Ostwald, 2001), such as the primary auditory cortex (A1). A1 neurons demonstrate reduced mean bandwidth in anesthesia than when animals are awake, with reductions up to threefold (Qin et al., 2003). In particular, ketamine anesthesia depth modulates not only average evoked responses but also response variability, which is highest under medium anesthesia, where ongoing cortical activity exhibits rhythmic bursting activity (Kisley and Gerstein, 1999). Importantly, this observed variability in shape and amplitude can be accounted for by the background ongoing activity, which speaks for transitions in thalamocortical excitability modulating these effects (Zurita et al., 1994). Specifically, stronger excitatory responses are observed in the thalamus after ketamine injection, despite decreasing overall cortical and thalamic firing rates (Kisley and Gerstein, 1999). Halothane, a gas anesthetic, shows a weaker suppressive effect on auditory-evoked responses (Johnson and Taylor, 1998; Moshitch et al., 2006), with responses found to sometimes resemble those in awake animals. Auditory working memory was found to be active for hundreds of ms after stimulus onset (Moshitch et al., 2006). Urethane causes moderate cardiovascular depression, with long duration of anesthesia (greater than 24 h), excellent anesthesia depth, and analgesia (Field et al., 1993). During urethane anesthesia auditory neurons show higher minimum thresholds, lower spontaneous firing rates, longer response latencies, and more frequent occurrence of tuning alterations, with stronger inhibition (Huang et al., 2013).

Because anesthesia facilitates experimental procedures, there are a multitude of deviance studies done in different species of anesthetized animals. Most of the studies have been carried out in rats (Ruusuvirta et al., 1998; Lazar and Metherate, 2003; Eriksson and Villa, 2005; Astikainen et al., 2006; Nakamura et al., 2011; Taaseh et al., 2011; Xu et al., 2014; Takahashi et al., 2015; Ahnaou et al., 2017; Parras et al., 2017; Rui et al., 2018; Cappotto et al., 2021), and mice (Ehrlichman et al., 2008; Chen et al., 2015; Duque and Malmierca, 2015; Duque et al., 2018; Lipponen et al., 2019), with a few studies in non-human primates (Uhrig et al., 2014), guinea pigs (Kraus et al., 1994; Christianson et al., 2014), gerbils (Bäuerle et al., 2011), and songbirds (Beckers and Gahr, 2012). These studies mainly report successful recordings of SSA or MMN-like responses in auditory cortices, especially under urethane anesthesia (Astikainen et al., 2006; Taaseh et al., 2011; Duque et al., 2015; Rui et al., 2018). Nevertheless, depending on the used anesthetic, higher-level deviance responses are attenuated or eliminated, despite retained low-level responses to deviant stimuli, as for example under ketamine anesthesia (Ehrlichman et al., 2008; Uhrig et al., 2016). Uhrig et al. (2016) anesthetized macaque monkeys with propofol and ketamine and presented a local-global auditory task during anesthesia. The authors observed no local deviance responses during light propofol sedation and deep anesthesia. By contrast, the global effect was preserved in core auditory areas bilaterally and the MGN, as well as in the anterior cingulate and prefrontal areas, albeit with diminished activations compared to wakefulness. During anesthesia, the global effect was reduced compared to wakefulness in all brain regions.

Thalamic SSA responses were recorded during ketamine anesthesia in gerbils (Bäuerle et al., 2011). In order to control for auditory cortical regulatory effects on subcortical regions, the authors pharmacologically inactivated cortical regions using muscimol, which preserves subcortical auditory processing. Interestingly, this led to a reduction of responses in the MGB of the thalamus of the anesthetized gerbil. The authors interpreted their findings as a demonstration that SSA in subcortical structures is mainly regulated by the descending corticofugal system, highlighting a more general function in information processing than just novelty detection. Finally, another interesting study in anesthetized zebra finches (Beckers and Gahr, 2012) used a switching oddball paradigm with naturalistic short-range contact zebra finch social calls, different to usual zebra finch background vocalizations. Birds were anesthetized with isoflurane gas, which produces behavioral and physiological effects through binding at multiple targets in the brain and central nervous system (binding to GABAa receptors and enhancing GABAergic inhibition; blocking glutamate release by binding to NMDA receptors), and shows similar effects on EEG as propofol (Purdon et al., 2015). Results indicate deviance processing in secondary, but not primary, cortices, suggesting that deviant events, more than just stimulating a larger part of a single sensory processing network, may activate a different network compared to standards, eliciting more widespread activity. It is worth noting that social calls are more complex than the pure tones generally used in the majority of oddball paradigms, and thus might recruit more complex predictive mechanisms.

### Conclusion

Overall, studies in humans and animals suggest that auditory predictions are reduced but may still be present in conditions of sedation and anesthesia. Interestingly, scalp EEG components corresponding to auditory predictive processes like the MMN or P3a may be preserved in anesthesia but are altered with respect to wakefulness. The latencies of scalp level auditory and deviance components are longer, and their amplitudes decrease. Moreover, the processing of deviant events at a local level is spatially restricted as shown via iEEG and source localization studies (Nourski et al., 2018; Zhang et al., 2018). Global deviance effects seem to be further restricted or even absent as the depth of anesthesia progresses in humans (Nourski et al., 2018; Shirazibeheshti et al., 2018), although they may be preserved in core auditory areas, at least in non-human primates (Uhrig et al., 2016). Importantly, similar to sleep, SSA is preserved also in anesthesia. These findings suggest that predictive processes are maintained to some degree under anesthesia, although they involve limited brain regions and sub-networks as compared to wakefulness.

## Disorders of Consciousness

One important application of auditory deviance paradigms has been the prognosis of patients with disorders of consciousness (DOC; Lew et al., 2003; Kotchoubey, 2005; Wijnen et al., 2007; Tzovara et al., 2013). DOCs are defined as a disrupted relationship between the two components clinically defining consciousness – wakefulness/arousal and awareness (Laureys, 2005). Coma is characterized by the absence of arousal and awareness. The vegetative state (VS) or unresponsive wakefulness syndrome (UWS; Laureys et al., 2010) is described by some degree of arousal in the absence of awareness, and the minimally conscious state (MCS) is characterized by preserved arousal with varying signs of awareness (Gosseries et al., 2011; Figure 1). In contrast, in the locked-in syndrome, often a consequence of brainstem damage, patients are fully aware and awake, but suffer from complete paralysis of all voluntary muscles except for vertical eye movements, as in amyotrophic lateral sclerosis (Bauer et al., 1979; Sharma, 2011). The famous American case of patient Terri Schiavo (see e.g., Perry et al., 2005) is a good example of the important and nuanced medical, ethical, religious, social, familial, philosophical, and political debates around retained awareness and prognosis in patients suffering from DOC.

About 50% of patients emerging from coma are expected to evolve into a MCS (Giacino et al., 2006), which is difficult to differentially diagnose from UWS because of intermittent signs of consciousness in MCS patients (Fins et al., 2007). Despite the immense societal importance, DOCs remain among the most poorly understood conditions of modern neurology (Boly et al., 2012). For many years, clinical and behavioral examinations were the leading approaches to diagnosing retained consciousness (Plum and Posner, 1982), but this approach has high rates of misdiagnosis (Laureys, 2005). Electrophysiology typically using ERPs is currently used in the majority of studies investigating patients with DOC (see Giacino et al., 2006; Owen and Coleman, 2007; Demertzi et al., 2008; Boly, 2011; Boly et al., 2012; Gosseries et al., 2014b), and is applied to the search for a “consciousness marker” to be used in diagnosis of DOC.

### Auditory Predictions and Their Link to Coma Outcome

Despite the heterogeneity of coma aetiologies and types of brain injury, several studies suggest that some patients in a coma can detect environmental deviant events at a neural level (Laureys et al., 2004; see also Table 3A for a summary). For instance, scalp EEG components such as the MMN and P300 correlate with patients’ outcome (Fischer et al., 1999; Kane et al., 2000; Luauté et al., 2005; Daltrozzo et al., 2007). Studies undertaken in the 90s have shown that some, but not all, coma patients may have preserved N100 (thought to reflect the early processing of acoustic features of a stimulus; Näätänen and Picton, 1987) and MMN responses, indicative of intact auditory deviance processing (Fischer et al., 1999; Daltrozzo et al., 2007). Interestingly, the presence of a MMN response was more frequently observed in patients who later awoke from coma (Fischer et al., 2004; Naccache et al., 2005), suggesting that the MMN is a predictor of patients’ chances of awakening. This hypothesis was driven by the fact that non-survivors generally did not show a MMN response (Fischer et al., 2004). However, these studies were performed several weeks or months after coma onset (Fischer et al., 2004; Boly, 2011).

**TABLE 3:** Studies in disorders of consciousness.

(A) Coma			

Study	Paradigm	Time of testing	Patients showing deviance effects
Fischer et al., 1999	Duration oddball	8.7 ± 11 days	33/128 patients
Fischer et al., 2004	Duration oddball	10.3 ± 11.4 days	88/346 patients
Luauté et al., 2005	Duration oddball	10.3 ± 11.4 days	Yes
Naccache et al., 2005	Pitch oddball	4–96 days	10/33 patients
Tzovara et al., 2013	Pitch, duration, location oddball	First 48 h	9/30 patients
Tzovara et al., 2015	Local-global	First 48 h	Global in 10/24 patients
Pfeiffer et al., 2018	Duration, location, pitch oddball	First 48 h	25/66 in 1st and 31/66 patients in 2nd day of coma
	Somatosensory oddball		16/66 patients in 1st and 23/66 in 2nd day of coma

**(B) UWS/MCS**			

**Study**	**Paradigm**	**Deviance effects**	**Patients showing deviance effects**

Perrin et al., 2006	Personal name oddball	Yes	6 MCS; 3/5 UWS; 4 LIS
Wijnen et al., 2007	Pitch oddball	Yes	10 UWS at first measurement
Bekinschtein et al., 2009	Local-global	Local	3/4 UWS, 4/4 MCS
		Global	3/4 MCS
Risetti et al., 2013	Pitch-duration oddball with own name; active counting of name	Yes	UWS: active < passive; MCS: passive > active
	Passive	Yes	10/11 patients
King et al., 2013	Local-global	Local	All
		Global	Only MCS, not UWS
Faugeras et al., 2011	Local-global	Yes	2/22 patients
Faugeras et al., 2012	Local-global	Local	Only CS and MCS
		Global	Only controls
Perez et al., 2021	Local-global	Local	N/A
		Global	43 (E)MCS/23 UWS out of 236 total

*N/A not reported; MCS, minimally conscious state; (E)MSC, (exit) MCS; UWS, unresponsive wakefulness syndrome; LIS, Locked-in Syndrome; CS, conscious ± paralysis.

More recent studies, performed in post-anoxic coma patients, have examined deviance processing in the acute coma phase, within the first 36 h of coma (Tzovara et al., 2013, 2016; Juan et al., 2016). In order to overcome the inherent difficulties associated with the detection of ERP components over single electrodes, these studies applied a multivariate decoding analysis (Tzovara et al., 2013) which models topographies of single-trial EEG activity. The model estimation was performed on a portion of the data (the training data set) and then used to decode the category of sounds (standard/deviant) in a separate portion of data. An above chance decoding performance implied a differential scalp EEG response to standard vs. deviant stimuli, focusing on the most discriminative time-windows within the trial. These studies have shown that during acute coma, even patients who do not survive show differential patterns of EEG activity in response to standard vs. deviant stimuli. Moreover, discrimination between standard and deviant sounds deteriorates from the first to the 2nd day of coma in non-survivors, while an improvement in auditory discrimination is observed for patients who later awake from coma (Tzovara et al., 2013, 2016).

More work in the acute coma phase using a local-global paradigm has shown that the global deviance effects, assessed via topographic patterns on scalp EEG, were preserved in 10 out of 24 patients (Tzovara et al., 2015). Moreover, while the global effect was not in itself predictive of the patient’s outcome, an improvement in decoding global standard vs. global deviant stimuli over the first 2 days of coma was informative of survival and return of consciousness (Tzovara et al., 2015).

The vast majority of deviance studies in coma target the auditory pathway, with the exception of one study comparing auditory and somatosensory stimuli, using the same oddball paradigm (Pfeiffer et al., 2018). Interestingly, this study found that discrimination between deviant and standard events at the EEG level is preserved in acute coma for both the auditory and somatosensory modalities. However, only the auditory modality was informative of coma outcome, with an improvement in auditory discrimination being indicative of survival. The specificity of deviance mechanisms for outcome prognosis is also highlighted by a study performed on the same type of patients, examining discrimination of naturalistic sounds, which, albeit preserved in some patients, was not informative of coma outcome (Cossy et al., 2014). Overall, these studies show that sensory deviance effects can be preserved in acute coma, suggesting a fundamental role for auditory predictions in relation to consciousness.

### Auditory Predictions Differentiating Consciousness Levels

Unresponsive wakefulness syndrome is typically characterized by spared brainstem activity with widespread severe damage to gray and white matter in both cerebral hemispheres (Laureys et al., 2004). Although brainstem metabolism can be spared in UWS, preserving arousal and autonomic functions, several cortical regions, including prefrontal regions, parietotemporal and posterior parietal cortices, and the precuneus, are typically impaired (see Laureys et al., 2004 for a detailed review). Regarding patients, spared medial parietal cortex (precuneus) and adjacent posterior cingulate cortex metabolism seem to differentiate MCS from UWS (Laureys et al., 2004). Overall cortical metabolism is slightly higher in MCS than in UWS patients (Laureys, 2005).

Deviance effects are posited to correlate with retained consciousness in UWS and MCS patients (e.g., Wijnen et al., 2007; see Table 3B for a summary). While MMN and P300 can be recorded in both clinical groups, global deviance effects in active tasks (e.g., counting the number of deviant stimuli, but without behavioral responses) are only recorded in MCS, and thus are associated with the presence of residual levels of consciousness. A study using a passive and active oddball paradigm (i.e., where participants had to count the deviant stimulus) in MCS and UWS patients recorded MMN (between standard and deviant tones) and P300 (in response to the patients’ own name) responses in all but one patient (Risetti et al., 2013). Nevertheless, only in MCS did the P300 increase in amplitude during the active condition, corroborating the possible advantage of using this paradigm for probing awareness by bypassing the motor response. In a similar paradigm, Perrin et al. (2006) observed the P300 response to patients’ own name in 3 out of 5 UWS patients, and in all MCS patients, concluding that this ERP component is not specific enough to differentiate UWS ad MCS patients.

When regularities are established over groups of sounds, past studies have shown a link between global deviance effects in UWS patients and the presence of residual consciousness (Faugeras et al., 2011, 2012; King et al., 2013). Particularly, global deviance effects have been linked to conscious perception, mainly supported by the absence of evidence for a global deviance effect in UWS patients (Bekinschtein et al., 2009; Faugeras et al., 2012; King et al., 2013). Bekinschtein et al. (2009) measured local deviance effects in UWS/VS and MCS patients, but no global effects. King et al. (2013), observed a global effect in 14 % of UWS and 31 % of MCS patients. A more recent study reported that the presence of a global deviance effect in UWS patients is related to an eventual return of consciousness, while its absence is not informative of patients’ outcome (Perez et al., 2021). In particular, the majority of patients that showed a global effect eventually regained consciousness, while amongst patients that did not show a global effect some regained consciousness, and some did not, paralleling findings based on MMN (Fischer et al., 2004).

When investigated during recovery from UWS, the MMN was found to be an important predictor of recovery ability (Wijnen et al., 2007), as MMN amplitudes increased with recovery. Moreover, a sudden increase in amplitudes preceding overt external communication was interpreted as consolidation of the networks and mechanism supporting this ability. The study of functional connectivity supports this hypothesis (Boly et al., 2011). Boly et al. (2011) used a roving MMN paradigm in MCS and UWS patients and modeled cortical source activity using scalp EEG data to quantify backward and forward connections between temporal and frontal cortices during MMN generation. The authors found that compared to MCS and healthy controls, UWS patients had impaired connections from frontal to superior temporal cortex, but no impairments in connectivity within temporal cortical networks.

### Conclusion

Taken together, studies in patients in a coma or with DOC show that scalp level EEG signatures of auditory predictive processes, including the MMN, may be preserved. An improvement of differential responses between standard and deviant stimuli over the 1st days of coma, or the presence of MMN responses in later coma stages, are frequently observed in patients that eventually regain consciousness.

Investigations of the neural circuits of predictive processes in patients with DOC remain sparse, and report that an impairment in predictive mechanisms may be accompanied by an impairment in backward connections from frontal to temporal cortical regions (Boly et al., 2011). One main challenge in studies with patients is pathological heterogeneity, for example relating to the cause of coma or DOC, to whether a focal lesion is present or not, or to the time of recording, as this may be followed by reconfigurations of brain networks supporting processing of environmental stimuli. Further studies of circuit level mechanisms are needed to better disentangle impaired and retained sensory predictive processes in patients with DOC, and link those to disease etiology and outcome.

## Altered States of Consciousness

Altered states of consciousness were first defined in the late 60’s as “any mental state(s), induced by various physiological, psychological, or pharmacological maneuvers or agents. An altered state of consciousness can be recognized subjectively by the individual […] as representing a sufficient deviation in subjective experience or psychological functioning from certain general norms for that individual during alert, waking consciousness” (Ludwig, 1966). Despite the fact that all the above-mentioned states can be considered altered states of consciousness, we here focus on those states induced by hypnosis and meditation (see e.g., Vaitl et al., 2005, for a review) due to availability of research using MMN paradigms in these states.

The psychological mechanisms that hypnosis and meditation engage are distinct: while hypnotic suggestions are utilized to elicit changes in experience, meditation may be considered as a form of mental training that induces alterations in attention and self-regulation (Jamieson, 2016). A common feature of hypnosis and meditation is that both processes involve self-regulation, including attentional control and self-awareness. These involve sensory and frontal-parietal attentional systems that also support predictive processing (Tang et al., 2015; Jamieson, 2016). The human brain is hypothesized to use both perceptual and active inference to maximize the effectiveness of predictive processing: for perceptual inference internal models are adjusted to best fit perception using predictions that best explain the experienced sensory information, whereas active inference consists of performing actions that produce sensory input conforming to predictions (Martin and Pacherie, 2019). Perception in itself can be divided into exteroception (perception of the external world), proprioception (perception of one’s own motion and one’s body in space), and interoception (perception of one’s own homeostatically regulated physiological states) (Jamieson, 2016), all of which are used to generate predictive models of the world, our bodies and our mental states. As discussed below, the processes of perceptual and active inference are altered during both meditation and hypnosis through modified priors as well as through altered perception. Despite sparse research into the topic of auditory deviance processing in hypnosis and meditation, the few existing studies are worth discussing, due to insights they might offer into mechanisms of self-regulation.

### Meditation

Meditation describes practices of self-regulation (Kabat-Zinn, 1982) and modulates the awareness component of consciousness (Brown and Ryan, 2004). Predictive processing during mindfulness meditation is thought to correspond to reductions in active inference and in the influence of priors (Pagnoni, 2019), as well as reduced stimulus salience weighting (Jamieson, 2016) – leading to reduced PEs, and thus reduced updating of expectancies, with parallel enhanced precision of proprioceptive and interoceptive predictions (Pagnoni, 2019). Collectively, these processes might lead to enhanced matching of interoceptive predictions and feedback (Jamieson, 2016), and thus to meta-awareness (Pagnoni, 2019).

Several ERP studies have investigated auditory oddball paradigms in mindfulness meditation (Cahn and Polich, 2009; Atchley et al., 2016; Biedermann et al., 2016; Fucci et al., 2018; see Table 4A for a summary). Cahn and colleagues compared a passive oddball task to a control thought period in expert meditators. They observed reduced amplitudes of the N1 and P2 components, representing early processing of acoustic features of a stimulus, and later P300 components to deviant tones and distractors (white noise), but not to standards (Cahn and Polich, 2009). Another study showed reductions in amplitudes of N1 and P2 components for all types of stimuli (standards, deviant, distractor), but not later P300, during mindfulness as compared to a tone detection task in expert and novice meditators versus controls (Atchley et al., 2016). A recent study in novice and expert meditators compared MMN responses during mindfulness meditation to a reading control condition (Biedermann et al., 2016). MMN amplitude was larger for both reading and meditation conditions in meditators as compared to controls. In novices, MMN responses were also increased during meditation as compared to reading. Taken together, these results indicate that mindfulness meditation might be associated with larger early sensory detection peaks for standard events, larger MMN responses and reduced P3a responses compared to normal wakefulness, which might be interpreted as greater environmental monitoring abilities, then applied to disengaging from distracting stimuli (supported by smaller early sensory detection peaks for distractors).

**TABLE 4:** Studies in altered states of consciousness.

(A) Meditation			

Study	Paradigm	When	Deviance effects
Cahn and Polich, 2009	Pitch oddball with distractor	During meditation	Yes
Atchley et al., 2016	Active pitch oddball with distractor	Before meditation	Meditators > controls
	Passive pitch oddball with distractor (meditation)	During meditation	Controls > meditators
Biedermann et al., 2016	Pitch oddball	Imaginative task	Meditators > controls
		During meditation	Meditators > controls
Fucci et al., 2018	Pitch oddball	Open presence meditation	Meditators = controls
		Focused attention meditation	Meditators > controls
		Reading	Meditators = controls

**(B) Hypnosis**			

**Study**	**Paradigm**	**When**	**Deviance effects**

Csépe et al., 1997	Phoneme oddball	During hypnosis	Hypnposis < baseline
Kallio et al., 1999	Pitch oddball	During hypnosis	Hypnosis > baseline (one virtuoso)
Jamieson et al., 2005	Roving standard	Before hypnosis	Yes
		During hypnosis	Yes
		After hypnosis	Yes
Hiltunen et al., 2019	Pitch oddball	Before hypnosis	Yes
		During hypnosis	Yes
		After hypnosis	Yes

### Hypnosis

Individuals who are susceptible to hypnosis are reported to experience changes in subjective awareness (Kihlstrom, 2005; Pekala, 2015). Hypnosis is thought to affect both active and perceptual inference, as well as perception, per se through attentional modulation (Jamieson, 2016, 2018; Martin and Pacherie, 2019). There are only a handful of studies investigating auditory predictive processes during hypnosis (Csépe et al., 1997; Kallio et al., 1999; Jamieson et al., 2005; Hiltunen et al., 2019; summarized in Table 4B). Perhaps the earliest systematic studies of this type were conducted by Gruzelier and colleagues (see Gruzelier, 1998, for a summary). In brief, medium-high hypnosis susceptible participants, but not low, showed decreased P300 to auditory oddballs and reduced MMN amplitudes during and following a hypnotic induction compared to pre-induction. By contrast, participants with low susceptibility showed an increase in MMN amplitudes following hypnotic induction. Measuring deviance responses in a passive oddball paradigm before the hypnotic induction and during neutral hypnosis (Kallio et al., 1999), as well as after the hypnosis in highly hypnotisable subjects (Jamieson et al., 2005; Hiltunen et al., 2019), and sometimes also using phonemes and participants with different levels of hypnotic suggestibility (Csépe et al., 1997), different studies demonstrate either increases or decreases of MMN amplitudes during hypnosis as compared to pre- or post-hypnosis. Another study found suppressed MMN amplitudes during hypnosis in highly hypnotisable subjects and no differences during waking between high, middle and low hypnotisable subjects (Csépe et al., 1997). While no changes were found in a recent study focusing on mean amplitude of ERP components from responses to standard and deviant sounds (Hiltunen et al., 2019), Jamieson et al. (2005) found increases in amplitude for MMN over frontal electrodes during hypnosis as compared to pre- and post-hypnosis in high suggestible participants (Jamieson et al., 2005). This trend was observed for these participants in temporal electrodes, too, but not for low suggestible participants, who showed linear increases in these electrodes from pre- to during to post-hypnosis. One possible interpretation for these results is that precision of deviance processing was enhanced, despite the engagement of attentional control with another active task.

### Conclusion

As a general conclusion, it is hypothesized that both meditation and hypnosis modulate predictive processes manifesting through scalp EEG components. For meditation, the results are too sparse and heterogeneous to draw firm conclusions, highlighting the need for more research. To address these heterogeneous results, predictive processing theories offer testable hypotheses to assess these changes in awareness and subjective perception that are at the core of these states. Some of the seemingly inconsistent results in hypnosis and meditation emphasize the limitations of this literature: the focus on analysis of ERP components at single electrodes, the heterogeneity of instructions, high inter-individual variations, and the differences in statistical analyses and dependent variables, making it difficult to draw consistent conclusions. Future research can address these issues by focusing on replication studies using similar task instructions, and moving beyond analysis of single EEG electrodes, to measures that quantify the whole electrical field at the scalp level (see e.g., Michel and Murray, 2012).

## Discussion and Future Outlook

A large body of literature has shown that sensory predictive signals manifest in the absence of consciousness. Here, we approached consciousness via states where consciousness is reduced or absent (sleep, anesthesia, disorders of consciousness), or altered (hypnosis, meditation). In the absence or alteration of consciousness, predictive processes can be preserved for predictions built over simple and long-lasting regularities. At the level of scalp EEG, evoked components associated with auditory predictions tend to have a reduced amplitude with decreasing levels of consciousness. At the level of generators, several studies suggest that the network underlying the generation of sensory predictions is restricted when conscious access and behavioral reactivity to the environment is lost. In the absence of consciousness, core auditory areas can preserve their capacities for generating deviance effects, while such effects in areas that are ‘higher’ in the sensory processing hierarchy (i.e., frontal areas) are abolished, likely as a result of disruption of connections from higher to lower regions.

However, as the generation of sensory predictions extends well beyond a two-node circuit of frontal-sensory areas, it remains an open question how each of the regions and the corresponding networks involved in sensory predictions is altered by the loss of consciousness. Importantly, the brain is a complex system, where mental states arise through the principle of emergence, and thus through an interaction of multiple functional, structural, and computational levels (Bassett and Gazzaniga, 2011). Within these computations, sensory predictive processes appear as a necessary, but not sufficient, condition for consciousness.

From an electrophysiological viewpoint, the loss of consciousness is accompanied by a plethora of changes in neural activity, such as the disruption of thalamo-cortical and cortico-cortical long-range connections, and changes in non-oscillatory components of the EEG (Magnin et al., 2010; Lendner et al., 2020, to name a few. These electrophysiological alterations may in turn affect circuit level mechanisms underlying predictions. Future studies should take into account these fundamental changes in neural activity when designing new experiments to study predictions in the absence of consciousness, and can choose to selectively stimulate specific states of neural activity, such as “up” or “down” sleep states.

In this review, we focused on neural signatures of predictive processes both at the neuronal level (e.g., SSA) and at the scalp EEG level (e.g., MMN or P300). The neural signals that can be recorded with scalp EEG have limited interpretation about the precise circuit or mechanisms underlying auditory predictions, because of the poor spatial resolution of EEG responses. Nevertheless, these scalp EEG components have strong clinical applications because of their relatively straightforward implementation (i.e., no invasive recordings are needed) that can facilitate their integration with other clinical measures to detect residual levels of consciousness.

### From Electrophysiology to Computational Models

As the loss of consciousness engenders drastic changes to the predictive circuit, another important future question is how these changes affect the neural computations that lead to a predictive signal. Although theoretical modeling has been widely applied in the field of threat predictions (e.g., Tzovara et al., 2018), or reward learning (Abbott and Dayan, 2005), attempts to model sensory predictions are limited. This is important given the fact that scalp EEG responses associated with deviance processing such as the MMN are compound responses, reflecting multiple and complex processes from multiple brain regions and neural computations. Distinguishing which neural computations of deviance processing (e.g., adaptation, PEs, update of an internal model) are performed in different cortical and subcortical structures involved in the sensory predictive network is a crucial future necessity.

Previous studies have tested various theories of auditory PE generation, and have shown that trial-by-trial changes in deviance EEG responses are compatible with a Bayesian updating of a probabilistic model of the environment in the auditory (Lieder et al., 2013), somatosensory (Ostwald et al., 2012), and visual modalities (Stefanics et al., 2018). Modeling work has also supported claims that deviance effects reflect PE signals, weighted by the precision of predictions (Stefanics et al., 2018), with attention increasing the precision of PEs (Smout et al., 2019). Nevertheless, the MMN still remains opaque in terms of which computational components it represents, and which changes these components undergo when consciousness is lost.

A principled way to model PE signals comes from the field of reinforcement learning (see e.g., Hoy et al., 2021). When studying reward PEs, past studies have applied an axiomatic model developed in the field of economics to assess whether responses indeed reflect PEs (Caplin and Dean, 2008; Rutledge et al., 2010). Developed for testing dopamine-related hypotheses, namely whether the firing rate of midbrain dopamine neurons reflect PEs, these axioms represent necessary and sufficient conditions for a brain response to be considered a true PE signal. Given theoretical work drawing similarities between reward and sensory PEs (Gardner et al., 2018), future studies can investigate computational approaches to offer more objective means to disentangle complex constructs such as the MMN.

Regarding the ambiguity as to which computational components are altered when consciousness is lost, some first attempts to resolve this question have used ketamine, which was shown to diminish model quantities that correspond to PE signals related to higher order predictions, like transition probabilities (Weber et al., 2020). Another recent study examined how awareness and task-relevance affect the neural computations of the MMN component (Schlossmacher et al., 2021). When stimuli were task-irrelevant, both in unaware and aware conditions, the MMN was best explained by an adaptation model, whereas when stimuli were aware and task-relevant, the MMN was best explained by a precision-weighted prediction error. Interestingly, although the trial-by-trial N100 amplitude of the EEG response to repeated tones in UWS patients has been shown to change (Kotchoubey et al., 2006), indicative of cortical learning, to date there are no attempts to formally model such changes. Future studies will need to link the electrophysiological alterations that are observed in sensory predictions during sleep, coma or anesthesia to computational models, in order to obtain a mechanistic understanding of the neural computations underlying sensory predictions in the absence of consciousness.

An important future question is whether the presence or absence of consciousness can be linked to specific computations that result in the generation of prediction signals. It has been proposed that one of the main functions of consciousness is the generation of internal representations from incoming sensory input (Kanai et al., 2019) so that we can act meaningfully on this input (Hohwy, 2012). Under standard predictive theories, the influence of PEs depends on their precision (Auksztulewicz and Friston, 2015; Kanai et al., 2015), and, as explained previously, this is the effect of attentional selection. This means that ascending PEs with higher precision have more model-updating power than those with lower precision (Witon et al., 2020). Future studies can evaluate whether a similar computational role can be attributed to different states of consciousness and, in particular, according to their arousal and awareness contents.

### Conclusion

In this review, we summarized studies investigating sensory predictions and their modulations by the loss of consciousness. We reviewed studies of animal and human physiology, from the fields of sleep, anesthesia, disorders of consciousness, hypnosis and meditation. Predictive processes represent a key, cross-species mechanism of perception, that manifests in an automatic way, and is embedded in distributed neuronal circuits. Refining our understanding of the neural networks and computations that underly sensory predictions in the physiological absence of consciousness (i.e., sleep or anesthesia) can advance our understanding of its pathological loss, and lead to improved, theory-driven strategies for diagnosis and prognostication in patients with disorders of consciousness.

## Author Contributions

RT, RK, and AT wrote sections of the manuscript. All authors read and approved the submitted version.

## Conflict of Interest

The authors declare that the research was conducted in the absence of any commercial or financial relationships that could be construed as a potential conflict of interest.

## Publisher’s Note

All claims expressed in this article are solely those of the authors and do not necessarily represent those of their affiliated organizations, or those of the publisher, the editors and the reviewers. Any product that may be evaluated in this article, or claim that may be made by its manufacturer, is not guaranteed or endorsed by the publisher.
